# Supplementary figures and images for: Dynamic causal modelling shows a prominent role of local inhibition in alpha power modulation in higher visual cortex
Source: PLoS Comput Biol. 2022 Dec 27;18(12):e1009988. doi: 10.1371/journal.pcbi.1009988 (PMC9829170; doi:10.1371/journal.pcbi.1009988)

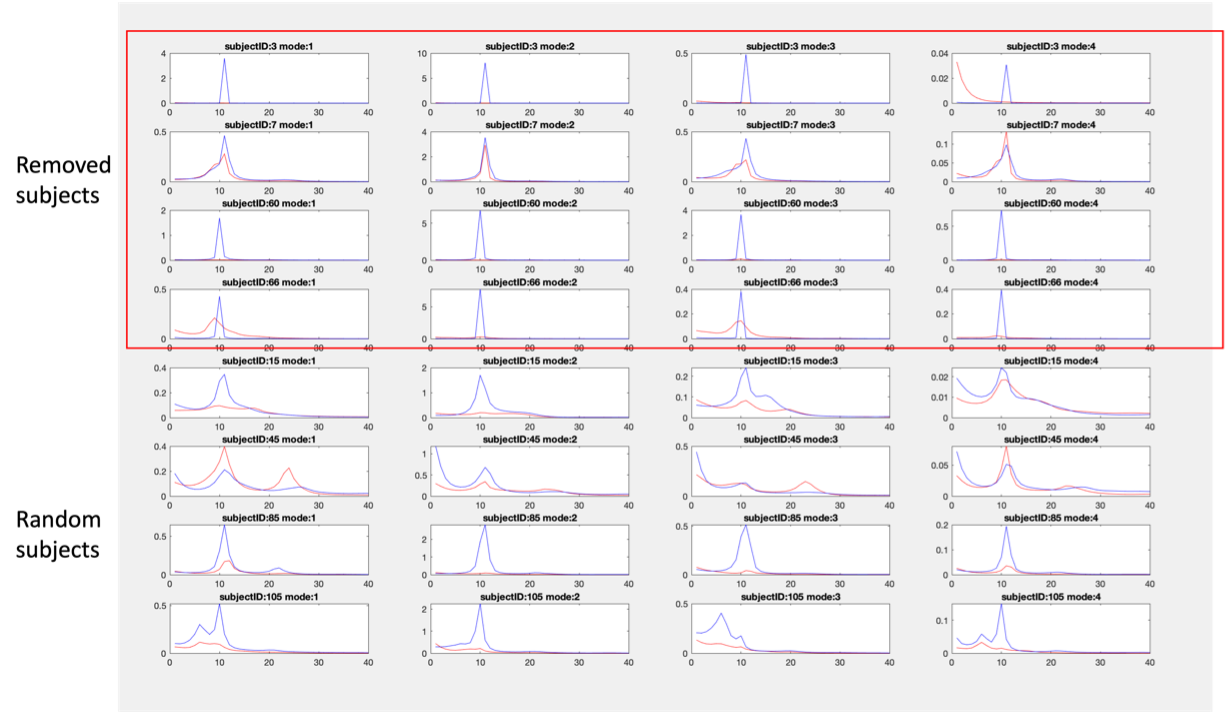

Supplement: S1 Fig — This figure displays the spectral data of the 4 data modes of the 4 removed subjects (top 4 rows) and 4 randomly selected subjects (bottom 4 rows). The 4 columns are the 4 data modes. Power spectra are shown for the EO (in red) and eyes closed (in blue) condition. The spectral densities were obtained by means of a Bayesian autoregressive model (see main text). (PNG) [file pcbi.1009988.s001.png]
