# Supplementary material for: Dynamic causal modelling shows a prominent role of local inhibition in alpha power modulation in higher visual cortex
Source: PLoS Comput Biol. 2022 Dec 27;18(12):e1009988. doi: 10.1371/journal.pcbi.1009988 (PMC9829170; doi:10.1371/journal.pcbi.1009988)
Supplement: S1 Appendix — This appendix summarizes the variational Bayesian framework used in this paper. (DOCX) [file pcbi.1009988.s002.docx]

## Variational Bayes model inversion

The variational Bayesian (VB) inversion scheme employed in this work can invert any (non-linear) model of the form:

$$y=f\left( \theta,u \right)+\epsilon$$

With $f\left( \theta,u \right)$ a (non-linear) function of the model parameters $\theta$ and possibly other known variables *u* (e.g. variables related to the experimental design, such as encoding the type of stimuli etc.) and $\epsilon$ is zero mean additive Gaussian noise with covariance $\Sigma_{y}$. Here $\theta$ denotes all the parameters of the model, so for a given DCM this entails all the parameters of the neural state equations and observation equations. The noise covariance is further decomposed in variance components with hyperparameters $\lambda$, which is explained in more detail further below. The likelihood of the data for model m is thus:

$$P\left( y\theta,\lambda,m \right)=N(y;f\left( \theta,u \right),\Sigma_{y})$$

Given the Bayesian nature of the framework, priors of model parameters ($\theta$) need to be specified and here has a (multivariate) Gaussian form:

$$P\left( \theta m \right)=N(\theta;\mu_{\theta} ,\Sigma_{\theta})$$

The error covariance is modelled as following:

$$\Sigma_{y}^{-1}=\sum_{i} exp(\lambda_{i})Q_{i}$$

Where $Q_{i}$ are known/assumed precision components (e.g. i=1 and $Q_{1}=I$ for uncorrelated white noise). The collection of hyperparameters $\lambda$ are also estimated during model inversion and have a Gaussian prior:

$$P\left( \lambda m \right)=N(\lambda;\mu_{\lambda} ,\Sigma_{\lambda})$$

The joint likelihood of a given model is thus:

$$P\left( y,\theta,\lambda m \right)=P\left( y,\theta,\lambda,m \right)P\left( \theta m \right)P\left( \lambda m \right)$$

And the full Bayesian model thus reads:

$$P\left( \theta,\lambda y,m \right)=\frac{P\left( y,\theta,\lambda,m \right)P\left( \theta m \right)P\left( \lambda m \right)}{P\left( y \right)}$$

$$P\left( ym \right)=\int\int P\left( y,\theta,\lambda,m \right)P\left( \theta m \right)P\left( \lambda m \right)d\theta d\lambda$$

Since the marginal likelihood $P\left( ym \right)$ involves an intractable integral, VB transforms the integral into an optimization problem by placing a lower bound on log model evidence using approximate posteriors which are assumed to factorize:

$$Q\left( \theta,\lambda m \right)=Q\left( \theta m \right)Q\left( \lambda m \right)$$

$$Q\left( \theta m \right)=N(\theta;m_{\theta} ,S_{\theta})$$

$$Q\left( \lambda m \right)=N(\lambda;m_{\lambda} ,S_{\lambda})$$

With $Q\left( \theta\right)$ and $Q\left( \lambda\right)$ the approximate posterior distribution of the model parameters and hyperparameters respectively. It can be shown that the log model evidence can be decomposed into:

$$\log P\left( ym \right)=F(m)+KL[Q\left( \theta,\lambda m \right) P\left( \theta,\lambda y,m \right) ]$$

Where the last term is the Kullback-Liebler (KL-) divergence between the true posterior and the approximate posterior. Since KL-divergence is always positive, it can easily be seen that the negative Free energy is a lower bound on the log model evidence with:

$$F(m)=\int\int Q\left( \theta,\lambda m \right)log\left[ \frac{P\left( \theta,\lambda y,m \right)}{Q\left( \theta,\lambda m \right)} \right]d\theta d\lambda$$

The parameters of the approximates posteriors and negative free energy can iteratively be estimated using the Laplace approximation. Note that the Laplace approximation provides an approximate negative free energy. It can furthermore be shown that the negative Free energy under the Laplace approximation can be decomposed into an accuracy term and complexity term:

Accuracy

Complexity

$$F_{l}\left( m \right)=-\frac{1}{2}e_{y}^{T}\Sigma_{y}^{-1}e_{y}-\frac{1}{2}\log\left| \Sigma_{y} \right|-\frac{N}{2}\log(2\pi)$$

$$-\frac{1}{2}e_{\theta}^{T}\Sigma_{\theta}^{-1}e_{\theta}-\frac{1}{2}\log\left| \Sigma_{\theta} \right|+\frac{1}{2}\log\left| S_{\theta} \right|$$

$$-\frac{1}{2}e_{\lambda}^{T}\Sigma_{\lambda}^{-1}e_{\lambda}-\frac{1}{2}\log\left| \Sigma_{\lambda} \right|+\frac{1}{2}\log\left| S_{\lambda} \right|$$

$$e_{y}=y-g\left( m_{\theta} \right)$$

$$e_{\theta}=m_{\theta}-\mu_{\theta}$$

$$e_{\lambda}=m_{\lambda}-\mu_{\lambda}$$

With N the number of data point. As can be seen from the above equations, the complexity term is not simply the number of parameters or hyperparameters. The algorithm that iteratively updates the parameters of the posterior distribution, maximizing the negative Free energy is described in detail in ^1^ and also see ^2,3^ for more information and is implemented in SPM12 in spm_nlsi_GN.m
