# Supplementary material for: Dynamic causal modelling shows a prominent role of local inhibition in alpha power modulation in higher visual cortex
Source: PLoS Comput Biol. 2022 Dec 27;18(12):e1009988. doi: 10.1371/journal.pcbi.1009988 (PMC9829170; doi:10.1371/journal.pcbi.1009988)
Supplement: S2 Appendix — These notes provide more details on the priors of the log-scaling parameter. (DOCX) [file pcbi.1009988.s003.docx]

# Notes on log-scaling priors

The priors on intrinsic connectivity modulation are about the log-scaling parameters (see table 1 of the main text). Because of the reparameterization, the actual scaling parameters follows a Log-normal distribution. To demonstrate this, we have simulated 10^5 random numbers from the (prior) normal distribution with mean = 0 and variance = .25. These numbers where then exponentiated and plotted in a histogram. Next, the corresponding log-normal distribution is overlaid with this empirical histogram together with the 5^th^ and 95^th^ percentiles.


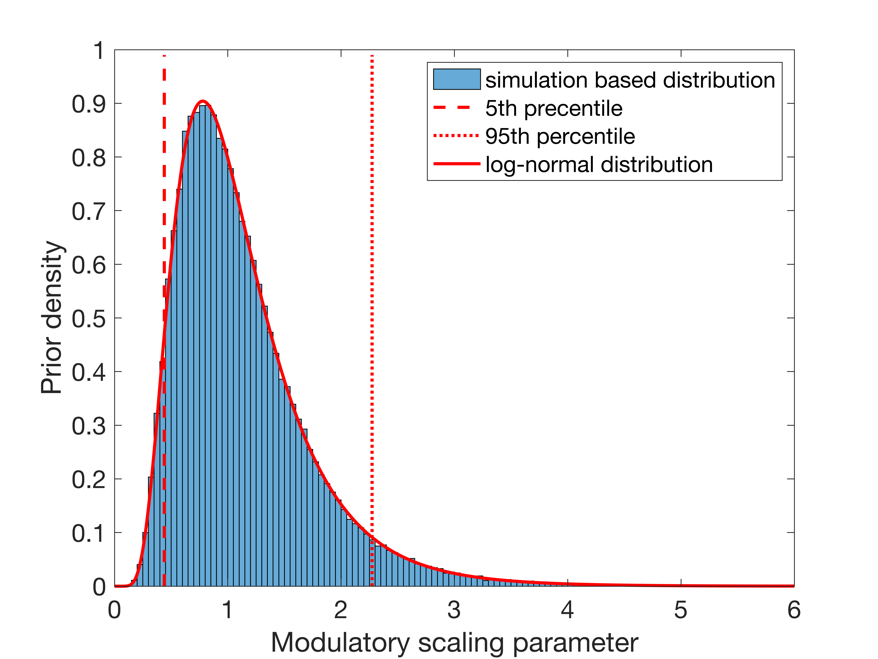


These percentiles can be treated as bounds on the plausible range of the scaling parameter (i.e. $G_{i}^{B}$falls between .44 and 2.29) and reflect halving and doubling baseline intrinsic connectivity following EC. The code for generating the figure can be found here <https://github.com/Frederikvdsteen/EO_OC_DCM/blob/main/Log_normal_priors.m>
