# Supplementary material for: Dynamic causal modelling shows a prominent role of local inhibition in alpha power modulation in higher visual cortex
Source: PLoS Comput Biol. 2022 Dec 27;18(12):e1009988. doi: 10.1371/journal.pcbi.1009988 (PMC9829170; doi:10.1371/journal.pcbi.1009988)
Supplement: Attachment — Submitted filename: Response_letter_R1.docx [file pcbi.1009988.s004.docx]

Responses to comments on manuscript

We would like to thank the reviewers and editor for their thorough comments on our paper ‘*Dynamic causal modelling shows a prominent role of local inhibition in alpha power modulation in higher visual cortex’*. We are grateful for their interest in our work. The recommendations have vastly improved the quality of our manuscript. We noticed that something went wrong during the conversion from word to PDF of the original submission which seems to be a bug in MS word when using cross references. We apologize for this mistake and have made sure all figures and references are correctly formatted in the revised manuscript. Below you can find our response to the comments and questions raised by the three reviewers.

Reviewer #1: This paper seeks to identify the physiological origin of increased alpha activity which occurs in (most) individuals upon closing of the eyes. Dynamic causal modelling is used to fit publicly available EEG data from over 100 subjects. The model is composed of microcircuits in the V1, right and left V5 regions, each of which consists of spiny stellate, superficial pyramidal, deep pyramidal, and inhibitory subpopulations. The hypothesis is that changes in alpha activity are due to changes in the connections between neuron subpopulations. By inferring the presence of these connections, specifically the intrinsic connections (within a region) and the extrinsic connections (between regions), and how the connection strengths are modulated when going from eyes-open to eyes-closed conditions, the authors conclude that much of the increase in alpha activity upon closing of the eyes can be attributed to the reduction in intrinsic inhibition activity between certain subpopulations.

Modulation of alpha activity upon opening or closing of the eyes is one of the most ubiquitous and repeatable features of the EEG. The conclusions presented here are significant for this reason alone and are potentially relevant to clinical and pharmacological applications.

Thank you for your appreciative comments.

The previous literature on the subject is described well and a detailed comparison is presented with the recent study published in this journal (Ref [43]), which also concluded that cortical inhibition was important in modulating alpha activity. An important advance is the use here of cross spectral densities. Can the authors comment on whether any of the parameters could have been estimated by modelling the power spectrum alone?

Thank you for this interesting comment. Yes, it is possible to invert a DCM based on the power spectrum of a single channel. However, the accuracy of parameter estimates would be reduced compared to inverting model based on the cross spectral densities used here. Originally, Moran et al (2009) only used the absolute cross spectral data for inverting a DCM. In Friston, 2012, the variational Bayesian inversion scheme was generalized to accommodate complex data. Friston et al 2012, showed using simulation that DCM-CSD with complex data features could more accurately recover ground truth parameters compared to the older absolute CSD based DCM’s. In addition, the posterior precision of some parameters increased for inversion of complex data compared to absolute CSD data. In summary, power spectral data of a single channel can be used to invert a DCM model but Friston et al 2012 clearly showed that complex CSD’s perform better because they have access to phase information and delays.

In general I found the work convincing although I did have to work through several of the references to get there.

I still have a few questions having to do with the data analysis.

(1) p.9 Line 203. “The first 4 eigenmodes of the prior data covariance are used to project the channel data into a reduced sensor space”. Does this mean that only data which is consistent with the model is being used in the fit? Approximately what proportion of the variance is discarded? I was not able to find much indication of this in Ref [40].

Thank you for the comment. The main reason why this approach is used in the current implementation of DCM is that this procedure furnishes a subspace which spans signals that can be explained by the observation model. This means that the reduced data space depends on the specification of the prior locations and the mapping between hidden source and sensor data. This also implies that the reduced data space does not depend on the neural model (for example a model with or without intrinsic connectivity modulation would in both cases result in the same reduced data space. Removing a source from the model entirely would change the reduced data space.).

We have calculated the reduced prior data covariance, and this explains about 90% of the full prior data covariance. In order to further investigate how much of the actual observed data is ‘used’ by the model, we perform the following analysis: For each channel, we used the 4 reduced data modes (in the temporal domain) to perform a linear regression, with the 4 reduced data modes as independent variable, and the channel time series as dependent variables. From this linear model, the R^2^ was extracted. This was performed for each channel and subject separately. Below, we show the results in image format. The x-axis are all 109 subjects, and the Y-axis are all EEG channels. The colorbar signifies the R^2^. We can see that the posterior electrodes (e.g., Pz P1-P8; PO7,PO3,PO8, O1, O2, OZ) can be very well explained by the 4 reduced data modes. In addition, the explained variance appears to be quite consistent across subjects.

In the figures below we used a topoplot and scatter plot to visualize the R^2^ for each channel averaged across subjects.

It is clear that the reduced data explains the posterior and central electrodes very well. The fronto-temporal electrodes are less well explained with R^2^ ranging between 0.4 and 0.6. Given that we focused on alpha power differences between eyes open and eyes closed, we are confident that the reduced data is adequate for the study.

(2) p.10. Table 1. I could not find where the coefficients specified for the different parameters (in the column “Parameterisation”) was explained. For example, where do the values [4,4,8,4,4,2,4,4,2,1] come from? How well known are they, and how might different values influence the conclusions? e.g. G3 has the largest coefficient and also has the largest (absolute) posterior mean value (Figure 6, top).

Most of priors on these parameter are the default parameters used in the vanilla DCM and are specified in previous papers that used the CMC model (see [1]). The only neural prior we had to define ourselves were the priors on the intrinsic connectivity modulations. Here, we used a relatively uninformative prior with a prior mean of 0 and variance of ¼. This means that connectivity modulation due to EC condition can be substantially increased (or decreased) by a factor of 2.3 (or 0.44). These values are obtained by exponentiating ±2 standard deviation of the prior: exp(±2*0.5) (taking the exponent is due to the re-parametrization explained in table1 of the main text).

In the text we have added the follow sentences:

*“*Most of the parameters and their priors provided in the table are the default priors of the DCM routine that was modified for implementing the intrinsic connectivity modulations (see spm_dcm_csd.m and spm_dcm_neural_prior.m and spm_fx_cmc.m of SPM12 version: 7487). The default priors were specified originally in [1] and where chosen to incorporate the known relative dissociation of the frequency content of superficial (high frequency) and deep pyramidal (low frequency) cells. Here, only the prior on the intrinsic connectivity modulations were custom specified and set to a prior mean and variance of 0 and 0.25 respectively. This can be seen as a relatively uninformative prior which allows for the intrinsic connection to be modulated substantially during EC. All the code for the analysis used in this paper are made publicly available at GitHub (https://github.com/Frederikvdsteen/EO_OC_DCM). “

Parameter G3 in figure 5 is indeed the largest effect but quite close to G1. Note that these reflect the connectivity modulations (i.e., parameters *G^B^_i=1,…10_ in table 1* ) not the baseline connectivity values (*G^A^_i=1,…10_* ).

(3) p.11. Line 218. “In total, 5 subjects were removed from the group analysis.” This represents ~5% of the subjects. Were there particular features of these discarded datasets that made the model fit inadequate?

Thank you for your comment. In fact, we made a small reporting error, there were only 4 subjects removed from the analysis leaving a total of 105 subject. Below we provide the auto-spectra of the 4 removed subjects and 4 subjects that were used for group inference.

The spectra of the EC condition (Blue lines) and EO condition (red lines) or shown for the 4 data modes. We can clearly see that the subjects that were removed, show a very prominent alpha peak, one that completely dominates the spectrum. For the spectra of the ‘good’ subjects, the alpha peak is prominent but doesn’t dominate entirely the spectra. In addition, these peaks appear a bit smoother compared to the alpha peaks of the ‘bad’ subjects.

We have added this figure in the supplementary materials.

I also suggest some minor corrections or clarifications:

p.7 Figure 1. “Intrinsic” is misspelled twice, next to the figure.

Thank you. Corrected

p.10. Table 1. The reference in the caption is misspecified.

Corrected

p.10. Line 213. Is the ‘IMG’-option that specifies the forward model an option in the code?

Yes, the alternative option is the ECD (equivalent current dipole), where a source is a dipole where dipole orientation is estimated. This option can be select in the code. In the code provided on GitHub, this option can be changed (to ‘ECD’) in ‘specify_DCM_models.m’, line 34:

p.13/14. Figure 3 is shown twice, once on each page.

Thank for the comment. As noted above, after submission we found out that something went terribly wrong during PDF conversion, which seems to be a bug in MS word when using cross references. We have modified this mistake.

p.16. Figure 5 is not referred to in the body of the text.

Thank you for pointing this out, this was accidentally removed from the manuscript: we added the following paragraph:

“The group analysis of the intrinsic connectivity modulation can be found in **Figure 5**. In V1, we observed a significant reduced of the inhibitory connection G_3_ (II → SS) and G_1_ (SS → SS). The excitatory connection G_5_ (SS → II) increased during EC. The pattern for left and right V5 are similar: reduced modulation of the inhibitory connection of G_3_ (II → SS) and G_1_ (SS → SS). and increased inhibition of G_9_ (II → DP).

“

p.17. Figure 6. The colour bar is labelled “dO/d\theta”. I could not find where “O” is defined.
p.17. Line 310. “Technically, we are numerically evaluating the Jacobian of the generative model of the extrinsic and intrinsic connectivity modulations at their posterior means.” I assume the output being evaluated is the power in a particular frequency channel? Is this “O” in Figure 6?

We apologize for not having made this clearer, “dO/d\theta” is indeed the power of the output at a particular frequency. We have adjusted the paragraph:

“Technically, we are numerically evaluating the Jacobian of the generative model with respect to the extrinsic and intrinsic connectivity modulations at their posterior means ($\frac{\partial O}{\partial\theta}$, with $O$ denoting the power spectrum) over the different frequency bins.”

And we have added the following sentence to the caption of figure 6:

$"\frac{\partial O}{\partial\theta}$ *is the Jacobian of the power spectrum of the generative model with respect to the model parameters of interest (intrinsic and extrinsic) which are shown for all modelled frequency bins.”*

p.17. Line 315. “Positive (red) and negative (blue) values indicate that increasing or decreasing the parameter would result in increased and decreased power, respectively.” The use of “respectively” is a bit confusing here. Can you separate this into 2 sentences?

We agree that this can cause confusion. We have replaced the sentence following your suggestion. This now reads:

“Simply put, positive (red) values indicate that an increase of a parameter at the posterior mean would result in increased power at that specific frequency bin. A negative value (in blue) means an increase of a particular parameter results in a decreased power.”

p.18. Line 342. “In two recent studies [43] and [43]”. Presumably one of these references should be [44]?

Indeed, we have corrected this mistake. Thank you.

-Damien Hicks

Reviewer #2: The study aims to understand the difference between eyes open and eyes closed brain states by inverting models of EEG. Unfortunately the manuscript lacks clarity and accuracy in presentation in a number of places, which makes assessing it difficult (see some details below). Also, part of the study using sensitivity analysis is limited to a single perturbation. More analysis is required there in order to support any conclusions.


clarity in introduction / motivation
- In the introduction it is stated that cortico-cortical interactions play more of a role than thalamo-cortical interactions. Could the authors please expand on the evidence for prominence of cortical interactions above thalamocortical interactions.

Thank you for this comment. We apologize for our wording. Our intention was not to claim that cortico-cortical interactions are more important than thalamo-cortical interaction. We have adjusted this sentence:

“….Moreover, it has been suggested that cortico-cortical interactions play a prominent role—in addition to thalamo-cortical dynamics—in the generations of alpha rhythms [9] and that they are associated with conscious perception [10]. ”

- in the motivation for dynamic causal modelling it is stated that measures like coherence "do not provide a unique or complete description of the underlying data generating process...". It is unclear what a complete description would be, what you mean by data generating processes, and in what sense dynamic causal modelling would provide a complete description of this. It would be good to clarify this, or give clearer motivation for the use of dynamic causal modelling.

Thank you for his comment. We agree that this sentence was not clear enough which was based on the following paragraph of Friston (2012, NeuroImage)), pg5:

“In summary both the real and imaginary parts of cross-spectra contain useful information about the underlying system. The modulus relates to the measure of coherence, while the argument (phase or angle) is a complicated function of phase-delays induced by exogenous fluctuations (innovations). However, neither provides a unique or complete description of how data are generated and may be better thought of as data features that have yet to be explained by a generative model.“

Our main point was that measures such as coherence do not provide a description of how data was generated (it lacks an observation equation). It only provides a measure of statistical dependencies among observed time-series. This is the major advantage of DCM; it provides a framework for estimating and comparing models of how neural units interact and map to observed responses. In this sense, measures such as coherence/cross correlation should be seen as data features. We have altered this sentence with the following:

*“However, previous work has emphasized that the modulus (coherence) or arguments (phase) of the cross-spectra densities alone do not provide a complete description of the underlying data generating process that produce spectral data features [17]. These measures only provide a description of the statistical dependencies between observed signals but not how they are generated by (hidden) neural states.”*

We modified the beginning of the next paragraph:

“One way to address some of these challenges is to use a forward (generative) model as in dynamic causal modelling (DCM). The generative model in DCM combines a biophysical and an observation model to describe the dynamics of hidden neural sources and how these neural states are translated into observed data. Furthermore, DCM provides a Bayesian framework to infer the unknown parameters of the model and to provide biophysical contraints on the neural dynamics. The biophysical model are sets of differential equations of coupled neural sources.”

- rather than specifically addressing the challenges laid out, isn't it rather the case that DCM would give complementary information?

It is usually argued that DCM and measures such as coherence (Granger causality etc.) have complementary agendas: DCM is a framework for the estimation of models of effective connectivity (i.e., generative models), while coherence is a measure of (directed- in the case of Granger causality) functional connectivity (see Friston, 2013).

Friston, K., Moran, R., & Seth, A. K. (2013). Analysing connectivity with Granger causality and dynamic causal modelling. *Current opinion in neurobiology*, *23*(2), 172-178.

- the authors should expand on in what sense DCM has been validated - this seems like a strong statement that potentially needs clarifying / caveating.

We agree that we should have been more thorough regarding the validity of DCM. We have replaced the sentence

“DCM has been validated, using intracranial and fMRI data, and has been shown to yield efficient source reconstruction [5].”

With the following:

“Several experimental studies in human and non-humans and simulation studies have shown the face validity [18], construct validity [19], predictive validity [20] and reliability[21] of DCM. Importantly, DCM for cross spectral densities was validated using an animal studies where DCM was able to correctly recover changes in synaptic physiology following neurochemical manipulation [22].”

- the statement that DCM yields efficient source reconstruction also should be expanded. Is that true for scalp EEG recordings of alpha rhythm, for example, is it true in general, or is this specific to the setup in reference 18?

We have removed this sentence about efficient source reconstruction to avoid confusion. We have replaced this sentence with several sentences about the validity of DCM (see previous comment.)

- "DCM .. provides a solution to the inverse problem.." Which specific inverse problem are you referring to?

We agree that this sentence was too vague. We are referring to the estimation of the hidden neural states that generated the observed data for a given estimated DCM. We have deleted this sentence to avoid confusion.

- ".. where both amplitude and phase information are used..." are you referring to phase information of a single signal, between signals or both?

The entire complex cross-spectrum is used during model inversion. So, the amplitude and phase of the auto and cross-spectrum of the data are used for model inversion. We have revised this sentence:

‘where both amplitude as well as phase information of the entire cross-spectra (including the autospectra) are used for inferring the underlying neural dynamics in terms of directed synaptic connections (i.e. effective connectivity). This means that phase information is also used in obtaining posterior estimates.’

- rather than only stating that the use of 3 distinct sources in their model is inspired by reference 8, the authors should give detailed reasons and evidence for why they made this assumption.

Thank you for his comment. We agree that we have gone over this too fast. In principle, it is known that the visual cortex is organized in such a way that information can travel through different occipital areas (e.g., visual information traveling from V1→V2→V3→V5 ). Due to the low spatial resolution of EEG data, sources that are located close to each other could generate almost the exact same data. As such, we assumed that adding V2,3 and V4 to our model would have resulted in an overly complicated analysis. In the discussion section we have also added the following paragraph:

“Using DCM, we specified a parsimonious model that can accommodate alpha power differences generated via changes in either within or between higher and lower order visual areas. Following [8], that found alpha traveling waves from higher to lower visual areas we considered three visual sources V1, left V5 and right V5. It is possible that other visual areas such as V2 and V4 as well the thalamus contributes to alpha rhythm generation. In our model, these contributions are modelled as spectral innovations with white and pink components; whose amplitude and slope are estimated for each brain area separately. Alternatively, one could implement Bayesian model comparison to identify the functional network, similarly to [65]”

clarity in methods:
- In the methods section and at the start of results there is reference to the permutation test that was carried out. For clarity, please make the description of this more explicit, and also include the motivation for doing this analysis. What t-test was performed exactly, i.e. distributions of which data were used? It is only stated that the EO and EC conditions were swapped.

We agree that we have not provided sufficient information regarding the non-parametric statistical testing. Our main motivation for doing this was to confirm the well-known effect that eyes-closed states are associated with increase alpha power compared to eyes-open states. We have added these sentences in the manuscript:

“permutation-based paired t-test was conducted by randomly assigning EO and EC labels to the entire power spectrum x channel data on a subject specific basis. We obtained 5000 permutations for the entire frequency x channel data space, during which the permuted T-values were retained. The T-value computed in each permutation was the same as the T-statistic that is used for a classical parametric paired T-test. The p-values were obtained by calculating the proportion of T-values obtained via permutation that exceeds the observed T-value, and the negative of the observed T-value. As such, a two-sided non-parametric paired T-test using permutation was performed. The major advantage of the permutation-based t-test is that it does not require any distributional assumptions about the test statistic.”

And

“Our first goal was to confirm the well-known effect on posterior alpha power: During wakeful state, eyes closed are associated with much greater alpha power compared to having the eyes opened.”

- The model needs to be described much more accurately and please be consistent with notation. Some examples: use of open circle and asterisk to denote multiplication (presumably) in different places; sigma only mentioned in the figure legend, and without giving its mathematical definition; no explanation of the biophysical meaning of the equations given; observation model not explicitly given

We have adjusted all the equations to ensure consistency. We have added the equations for sigma and have added the equations for the observation model:

“The forward model used here (the ‘IMG’-option), treats each source as a patch on the cortical surface [46]. More specifically, the form used here is:

$$y_{full}=g\left( x,\theta_{obs} \right)$$

$$g\left( x,\theta_{obs} \right)=\sum_{K=1:3} \Delta^{K}\sum_{j=1:8} \Psi_{j}x_{j}^{k}$$

$$\Delta^{K}=\sum_{n=1:6} {\Theta_{n}^{K}\Upsilon}^{K}$$

Here $y_{full}$ denotes the full EEG data and $g\left( x,\theta_{obs} \right)$ that observation model. $\Delta^{K}$ is a Laplacian operator of region K, that is modelled as a mixture of *n=1,..,6* spatial basis functions. These are obtained by taking the first 6 eigenvectors of the lead-field matrix of all the sources that fall within a sphere of 18mm of the cortical patch defined around the MNI coordinates of the 3 regions. These coordinates are: [0 -88 4] for V1 and [-44 -68 0] and [42 -72 0] for left and right V5, respectively. Here the lead-field matrix was created using a default head model implemented in SPM12. $\Theta_{n}^{K}$ are the spatial parameters that are estimated during model inversion. The term $\sum_{j=1:8} \Psi_{j}x_{j}^{k}$ quantifies the contribution with weights $\Psi_{j}$ of the neuronal populations $x_{j}^{k}$ to the EEG data. The weights are free contribution parameters that are also estimated during inversion. Note that here only the depolarizing voltages of SP, DP and SS are allowed to contribute $y_{full}$ and are the same across the three regions. $\theta_{obs}$ denotes all the parameters of the observation model that are estimated during model inversion (i.e. the collection $\Psi_{j}, \Theta_{n}^{K}$). The DCM for CSD was inverted using a variational Bayesian inversion scheme described in [47] and also see S1 Appendix for more details. “

In addition, we have added supplementary materials regarding the Variational Bayesian inversion scheme.

- The inference methods should be more accurately described, for example: no definition of any of the thetas is given; there appears to be inconsistency in subscript between thetas ("Y" or "G", what do these mean?); no motivation is given for the parameter ranges; for readers of plos CB at least some details of the inference scheme should be given.

We have added supplementary materials regarding the Variational Bayesian inversion scheme. Most of the priors on these parameter are the default parameters used in the vanilla DCM and were set in previous papers that used the CMC model (see [1]). The only neural prior we had to define ourselves were the priors on the intrinsic connectivity modulations. Here we used a relatively uninformative prior with a prior mean of 0 and variance of ¼. This means that connectivity modulation due to EC condition can be substantially increased are decrease. We have added several sentences clarifying the re-parametrization that is given in table 1:

*“*Most of the parameters and their priors provided in table 1 are the default priors of the DCM routine that was modified for implementing the intrinsic connectivity modulations (see spm_dcm_csd.m and spm_dcm_neural_prior.m and spm_fx_cmc.m of SPM12 version: 7487). The default priors were specified originally in [1] and where chosen to incorporate the known relative dissociation of the frequency content of superficial (high frequency) and deep pyramidal (low frequency) cells. Here, only the prior on the intrinsic connectivity modulations were custom specified and set to a prior mean and variance of 0 and 0.25 respectively. This can be seen as a relatively uninformative prior which allows for the intrinsic connection to be modulated substantially during EC. All the code for the analysis used in this paper are made publicly available at GitHub. (https://github.com/Frederikvdsteen/EO_OC_DCM). Also note that in table 1, the parametrization column indicates how the parameters in the right column are transformed into parameters that enter the neural state equations. This re-parametrization allows for sign constraints so that—for example—time constants are always positive. The parameters in the right column are those that effectively enter the model inversion scheme and here we used the ‘$\theta$*’* notation. For the re-parametrized parameters (left column) that enter the neural state and observation equations (see below), we used the notation that is also used in the SPM12 implementation. In S1 Appendix we provide more information regarding the variational Bayesian inversion framework. “

clarity in results:
- figure 2: it might be old fashioned, but I find referring to components of figures in order from left to right helpful. labels a, b etc can also help. Consistency is important - we have channels numbered or given their letter/number labels. Please alter so we can compare the two components of figure 2. Are there units for power?

Thank you for the comment. We have adjusted the figure, including letters for the different components of the figure, and have replaced the labels with number of the topographical figure. The units for power are now included (dB/frequency).

figure 3 is actually repeated twice in my pdf. Please explain more explicitly what you mean by taking all combinations of parameters.... What is the Null model?

As noted above, we discovered that something went very wrong with the cross-references during PDF conversion, which caused a duplicate of figure 3 and other referencing error. This is fixed in the revision. We have clarified further what we mean with taking ‘all combination of’ and the ‘Null model’. We have adjusted the manuscripts at several locations:

“As a first step, we created 8 alternative PEB models to test which connectivity modulations are related to the difference between EO to EC conditions. The 8 PEB models were created by taking the combinations of the following three parameter sets: with or without extrinsic modulations, with or without V1 intrinsic modulations and/or with or without V5 intrinsic connectivity modulations. This also includes a Null model with no extrinsic nor intrinsic connectivity modulations.”

And the caption of figure 3 was revised:

**“Figure 3.** **PEB model selection.** The bar graph of the free energy (i.e., log evidence) differences from the full model (i.e. the model with extrinsic, V1 and V5 connectivity modulations) are shown for the 8 PEB models considered. The models were formed by creating combinations with or without the following three parameters sets: extrinsic modulations (Ext), V1 intrinsic modulations (V1) and V5 intrinsic modulations (V5). We observe that the full model has the highest (approximate) model evidence. In addition, models without V5 intrinsic modulation have smaller evidence compared to models that included V5 intrinsic modulation.”

- Models without V5 have lower evidence - is this because there are more parameters in v5 as there are two of them? Please provide the formula for model evidence.

As requested above, we have added information about model inversion in the supplementary materials. We also provided the decomposition of the approximate log model evidence in terms of accuracy and complexity trade off where the complexity term does not simply reflect the number of parameters:

$$F_{l}\left( m \right)=-\frac{1}{2}e_{y}^{T}\Sigma_{y}^{-1}e_{y}-\frac{1}{2}\log\left| \Sigma_{y} \right|-\frac{N}{2}\log(2\pi)$$

$$-\frac{1}{2}e_{\theta}^{T}\Sigma_{\theta}^{-1}e_{\theta}-\frac{1}{2}\log\left| \Sigma_{\theta} \right|+\frac{1}{2}\log\left| S_{\theta} \right|$$

$$-\frac{1}{2}e_{\lambda}^{T}\Sigma_{\lambda}^{-1}e_{\lambda}-\frac{1}{2}\log\left| \Sigma_{\lambda} \right|+\frac{1}{2}\log\left| S_{\lambda} \right|$$

$$e_{y}=y-g\left( m_{\theta} \right)$$

$$e_{\theta}=m_{\theta}-\mu_{\theta}$$

$$e_{\lambda}=m_{\lambda}-\mu_{\lambda}$$

Here $F_{l}\left( m \right)$ is the approximate log-model evidence under the Laplace approximation (see supplementary materials.). In the second row, we can see the complexity terms that are related to the parameters (third row is the complexity term due to the hyperparameters), which can be seen as a proxy for the KL divergence (Prior$| |$ Posterior). In other word, if the distance between the prior and the posterior is large, complexity will be large. For example, it can be the case that a single parameter penalizes more when the deviation is substantial compared to the case when several parameters do not deviate from their prior (and hence have small penalty).

Complexity

Accuracy

- Please provide some exemplar fits so that we can see visually what the tradeoff between complexity and match to data is (presuming that is in the measure of evidence).

Below you can find some exemplar fits of the spectral power of the 4 data modes of 4 subjects. The rows are the different subjects while the columns are the 4 different reduced modes. Full lines are the predicted power by the model, while dashed lines are observed spectral data. In red are spectra during EO and in blue during EC.

- Figure 4: how does the parameter estimate relate to any of the Gs or thetas etc? Please be explicit. What is the posterior probability? Probability of what exactly?

Thank you for this comment. We agree that we were not sufficiently clear about what is shown in Figure 4. What is shown are indeed the thetas, but we labelled them with their parameter labels that are used in the equations (i.e.,. left-column of table 1.).

The thetas are used to denote all parameters that are estimated using the variational Bayesian inversion scheme. Some of these theta’s are transformed by the equations (see table 1) The G’s in figure 1 were used to label the within region coupling. In figure 4, we used explicit labels to denote the extrinsic connections (the dashed lines in figure1). We adjusted the figure caption including the following sentence:

“Note that the estimated parameters are log-scale parameters (i.e., the parameters of the right column of table 1 (*θ_B_*), but here we used different x-axis labelling for clarity.). The right panel shows the posterior probabilities of the extrinsic connections.”

In the methods section we have expanded a sentence to more clearly described posterior probability:

“Inference of second level parameters—encoding group-mean intrinsic connectivity modulations—used the posterior probability (Pp), that quantifies whether a model including this parameter explains the data better than a model without. This has the advantage that the full parameter covariance matrix is used when comparing models, see [27] for details. ”

- rV5->V1 has uncertainty encompassing zero, so is it true to say that all extrinsic connections increase?

You are right, we have reformulated this as follows:

“We observe that the forward extrinsic connections from V1 increase during EC. The backwards connections appear to increase as well, however, the evidence is not conclusive (Pp<0.95).”
- it seems figure 5 is not discussed in the text at al

Thank you for pointing this out, this was accidentally removed from the manuscript: we added the following paragraph:

‘The group analysis of the intrinsic connectivity modulation can be found in **Figure 5**. In V1, we observed a significant reduced of the inhibitory connection G_3_ (II → SS) and G_1_ (SS → SS). The excitatory connection G_5_ (SS → II) increased during EC. The pattern for left and right V5 are similar: reduced modulation of the inhibitory connection of G_3_ (II → SS) and G_1_ (SS → SS). and increased inhibition of G_9_ (II → DP).’

- why is e^-6 chosen as an increment? How does this result change if different values are chosen? What are the posterior parameter values that are fixed? Is there a simple explanation for the changes being largest in certain parameters in figure 6, such as the perturbation made to them is larger on the scale of their values? What if you make proportional changes? Please provide axis labels. What is dO/dtheta? "changes in intrinsic connectivity have a larger effect.." this is true for V5 but not for V1.

Thanks for the comment. If one wants to evaluate the partial derivates/Jacobian numerically, a specific number must be chosen quite arbitrarily as an increment. Here, we simply report the default increment that is used in the spm_diff.m function of the SPM12 software that is used for evaluating partial derivatives/Jacobians. This is the appropriate step size implied by the numerical integration scheme used in SPM. We perturbed each parameter around its posterior mean. We have added the following sentences:

“More specifically, we added a small increment ($e^{-6}$) to the posterior mean of a certain parameter, while keeping the posterior means of the other parameters fixed. This was repeated for all the parameters of interest separately. In total, we performed the sensitivity analysis at the posterior means of 33 parameters.”

We also revised the caption of Figure 6:

**Figure 6. Sensitivity analysis.** The results of the sensitivity analysis—averaged across subjects—are shown in image format. The group BMA results (i.e. the middle panels of **Figure 4 and 5**) are provided in the lower part for comparison. Simply put, positive (red) values indicate that an increase of a parameter at the posterior mean would result in increased power at that specific frequency bin. A negative value (in blue) means an increase of a particular parameter results in a decreased power. The arrows indicate the sensitivities for the significant intrinsic connectivity modulations that are shown in the lower part of the figure. $\frac{\partial O}{\partial\theta}$ is the Jacobian of the power spectrum of the generative model with respect to the model parameters of interest (intrinsic and extrinsic) which are shown for all modelled frequency bins.

Because we used log normal scaling priors, the perturbation for each parameter is the same in the scale of their values. In table 1, the increment is added to the parameter in the right column, but these are then exponentiated before entering the state equations. Moreover, we see both large and small perturbation effects: for example, the posterior mean of G_9_ in V5 is relatively large and the effect of a small increment is also large. On the other hand, the posterior mean of parameter G_6_ in lV5 is relatively small yet the increment has a quite large effect.

Discussion
- line 342: reference 43 repeated.

This was corrected, thank you.

Reviewer #3: This was a well described study using canonical microcircuit modelling and DCM to investigate intrinsic and extrinsic connectivity changes that may underlie the differences in alpha oscillations between eyes open and closed resting state. The study is well thought out and illustrated. My comments are generally minor.

I must note also that there are numerous formatting errors that while they won’t influence the final product after typesetting and editing, it made the paper unnecessarily difficult to read/review. This included numerous errors with in text referencing for tables and figures. Figure placement was problematic (sometimes half way through its own caption) see Results point 2 and 3. There was inconsistent referencing styles (number format and author date) as well as references to the same paper twice where there should have been two separate references.

Thank you for the comment. As noted above, after submission we found out that something went terribly wrong during PDF conversion, which seems to be a bug in MS word when using cross references. Our sincere apologies for this. We have adjusted the revised manuscript accordingly.

Methods
1. Why do you run a 60 Hz filter if you then go on to high pass filter the data at 45 Hz?

We used the standard pre-processing procedures which usually performs line-noise filtering in addition to broadband filter. I guess the line-noise filtering here was obsolete given our high-cut filtering at 45Hz.

2. Where you describe using the model the ‘canonical microcircuitry’, it would be useful to describe exactly which model you used. If you used the standard CMC out of the spm toolbox please describe the function you used that determines the model, and the SPM toolbox version. Then clearly refer to the modifications and relevant papers – which already do emerge in the following section. It would be ideal to be able to clearly evaluate this while reading to both smooth comparisons between DCM papers and any replication attempt.
(I did note afterward the code is available on GitHub and the implementation is clearly described in the ReadMe - so referencing that this is the case in text would also help).

I agree, I added the following sentence

“We used the spm_fx_cmc.m function that implements the equations described in Figure 1.”

In addition, we added a sentence referring to the GitHub page were al the code can be found:

“Most of the parameters and their priors provided in the table are the default priors of the DCM routine that was modified for implementing the intrinsic connectivity modulations (see spm_dcm_csd.m and spm_dcm_neural_prior.m and spm_fx_cmc.m of SPM12 version: 7487).”

3. For Figure 1, in the caption it would be useful to refer to Table 1 for complete interpretation of the equation notation, or describe/link this all up more clearly in the following section, or clearly reference the relevant papers.

We agree that we should have made clearer the link between figure 1 and table 1. We have added the following in the manuscript:

“Also note that in table 1, the parametrisation column indicates how the parameters in the right column are transformed into parameters that enter the neural state equations. This re-parametrization allows for sign constraints so that for example, time constants are always positive. The parameters in the right column are those that effectively enter the model inversion scheme and here we used the ‘$\theta$*’* notation. For the re-parametrized parameters that enter the neural state and observation equations (see below), we used the notation that is also used in the SPM12 implementation. In S1 Appendix we provide more information regarding the variational Bayesian inversion framework.”

4. Fix referencing error in Table 1: “Table 1. Parameters of neural model (see Error! Reference source not found. for illustration of the neural model)”

Thanks, we fixed all the reference errors

Results
Results were clearly presented and well furnished with figures – really just a couple of notes that impeded reviewing and ought to be fixed to help any further revisions:
1. Duplicate Figure 3 appears in text in section “PEB model selection and parameter averaging”. This appears to be related to an error in the way automatic figure referencing is happening in text.

Indeed, the Word bug cause some weird referencing errors and duplication of figures. This problem is resolved in the revised manuscript

2. Figure 6 appears halfway through your figure caption

This is again related to the pdf conversion bug.

3. Often the figures appear to randomly be placed mid paragraph, sometimes before their first reference in text.
This is again related to the pdf conversion bug.
Discussion
1. Duplicate references means one is probably missing? Line 342: “In two recent studies by [43] and [43]”

Thanks for pointing this out, we have corrected this.

2. Optional, but is it worth commenting on the implication of comparing EC vs EO data between clinical/pharmaco studies based on the mechanistic differences described here?

Thank you for the suggestion. We have added the following paragraph in the discussion:

“The clinical relevance of comparing eyes-open vs. eyes-closed data using DCM can be appreciated knowing that the (normalized) difference between EC and EO (which is sometimes termed alpha-reactivity) is related to specific neurological conditions [31, 57, 58]. However, reduced alpha reactivity can manifest itself in different ways. The reduction in reactivity can be due to increased EO alpha power and unaltered EC power, or decreased EC power and unaltered EO power. For example, [57] showed that in Alzheimer disease patients, EC alpha was reduced while EO alpha was not compared to healthy controls. The reverse was true for Lewy body dementia patients. Using DCM, one could identify which parameter of the model employed here could explain these observed ‘alpha reactivity’ differences. These parameters could possibly be useful as an aiding tool for differential diagnosis. “

3. Also just a suggestion, but given the paragraph starting “Given the role of inhibitory intrinsic connections found here,…” directly describes the study data and its impact/implications should it be the second paragraph of the discussion? At the moment it’s rather buried.

This section may be of future use to clinical and pharmaco resting studies. It will also be the section where the people who don’t understand/use DCM and canonical microcircuits will learn how to interpret the model findings in neurobiological terms.

Thank you for the suggestion and we agree, we have put this paragraph as second paragraph in the discussion.

4. What do you mean by innovations in the paragraph on 1/f fluctuations? Do you mean innervation?

Thank you for pointing to this. By innovations, we mean endogenous fluctuations that drive the system. The term “innovations” is also used to describe the noise in autoregressive models (<https://en.wikipedia.org/wiki/Autoregressive_model>).

We hope these revisions are what you and the editor had in mind.
