# Supplementary material for: Dynamic causal modelling shows a prominent role of local inhibition in alpha power modulation in higher visual cortex
Source: PLoS Comput Biol. 2022 Dec 27;18(12):e1009988. doi: 10.1371/journal.pcbi.1009988 (PMC9829170; doi:10.1371/journal.pcbi.1009988)
Supplement: Attachment — Submitted filename: Response_letter_R2_submitted.docx [file pcbi.1009988.s005.docx]

Responses to comments on manuscript R1.

We would like to thank the reviewers and editor again for their time and effort reviewing the revised manuscript. You can find our response to the remaining comments below. In addition, we have noticed a small reporting error in the formulation of the intrinsic connectivity modulation. Due to the rescaling of the parameter, the intrinsic connections are calculated using the following:

$$G_{i}=G_{i}^{A}G_{i}^{B}$$

$$G_{i}^{B}=exp({X\theta}_{Gi}^{B})$$

Where $G_{i}^{B}$is a modulatory gain parameter. We have adjusted the text accordingly (see also our response to Reviewer #2 for the full revision of this paragraph). Importantly, this small mistake does not influence any of the results and conclusions presented in the manuscript.

*Reviewer #2: Many thanks for the thorough revision. The manuscript is much more accessible now and I think it is suitable for publication. There are just two minor points that i think should be addressed and a few typos that i noticed:*

Thank you very much.

1. line 227- page 10. Can you give a figure in the manuscript to explain / quantify what "relatively uninformative" prior refers to? Perhaps you could show a plot of the prior distributions in supplemental so the reader can assess this for themselves?

This is a very interesting suggestion. We agree that due to the transformations being applied to some of the parameters, it is difficult to have an intuitive grasp about these priors. We have explained this in more detail and replaced the part about the re-parametrization for clarity. The full paragraph is now:

“In order to infer condition dependent changes in intrinsic coupling, the current DCM implementation of the CMC model described above was supplemented with parameters encoding these changes as following [25]:

$$G_{i}=G_{i}^{A}G_{i}^{B}$$

$$G_{i}^{B}=exp({X\theta}_{Gi}^{B})$$

Here, X encodes the conditions so that X = 0 for EO and X = 1 for EC condition. This implies that $G_{i}^{A}$ encodes baseline intrinsic connectivity and here corresponds to the EO-state. Consequently, $G_{i}^{B}$ encodes the modulatory gain of the *i-*^th^ intrinsic connection associated with the EC-state. In other words, the baseline intrinsic connections are rescaled by a factor equal to $G_{i}^{B}$. Connectivity and other parameters of the neural model are shown in Table 1. Note that in Table 1 the parametrisation column indicates how the parameters in the right column are transformed into parameters that enter the neural state equations. This re-parametrization allows for sign constraints so that, for example, time constants are always positive. The parameters in the right column are those that effectively enter the model inversion scheme and here we used the ‘*θ’* notation. For the re-parametrized parameters (left column) that enter the neural state and observation equations (see below), we used the notation that is also used in the SPM12 implementation. In the supplementary materials we provide more information regarding the variational Bayesian inversion framework. Most of the parameters and their priors provided in table 1 are the default priors of the DCM routine that was modified for implementing the intrinsic connectivity modulations (see spm_dcm_csd.m and spm_dcm_neural_prior.m and spm_fx_cmc.m of SPM12 version: 7487). The default priors were specified originally in [46] and where chosen to incorporate the known relative dissociation of the frequency content of superficial (high frequency) and deep pyramidal (low frequency) cells. Here only the prior on the intrinsic connectivity modulations were custom specified and set to a prior mean and variance of 0 and 0.25 respectively. This can be seen as a relatively uninformative prior which allows for the intrinsic connection to be modulated substantially during EC. Due to the re-parametrization, these priors are about log-scaling parameters and therefor the actual scaling ($G_{i}^{B}$) follows a Log-normal distribution (see supplementary materials S3 Appendix). The 5^th^ and 95^th^ percentile of this Log-normal distribution are .44 and 2.29 respectively. These bounds reflect halving and doubling baseline intrinsic connectivity. All the code for the analysis used in this paper are made publicly available at github (https://github.com/Frederikvdsteen/EO_OC_DCM). “

In the supplementary materials you can find the following:

“The priors on intrinsic connectivity modulation are about the log-scaling parameters (see table 1 of the main text). Because of the reparameterization, the actual scaling parameters follows a Log-normal distribution. To demonstrate this, we have simulated 10^5 random numbers from the (prior) normal distribution with mean = 0 and variance = .25. These numbers where then exponentiated and plotted in a histogram. Next, the corresponding log-normal distribution is overlaid with this empirical histogram together with the 5^th^ and 95^th^ percentiles.

These percentiles can be treated as bounds on the plausible range of the scaling parameter (i.e. $G_{i}^{B}$falls between .44 and 2.29) and reflect halving and doubling baseline intrinsic connectivity following EC. The code for generating the figure can be found here <https://github.com/Frederikvdsteen/EO_OC_DCM/blob/main/Log_normal_priors.m> “

2. line 389 page 16. It is stated that the largest negative sensitivities are for G3 and G1. It is qualified that you are looking at the significant intrinsic modulation, but it is worth pointing out and explaining that the largest sensitivities are actually for G4. It's interesting that this parameter can cause large deviations in the spectrum but has not been identified as an important parameter in the DCM.

This is indeed an important point. We have added the following lines:

“Interestingly, the inhibitory G_4_ (II → II) connections in V5 seem to have the largest sensitivities as well but are not significant parameters in the PEB analysis. This suggest that a reduction in G_4_ in V5 could potentially be important for enhancing alpha power. Nevertheless, the current parameter configuration appears to suffice (in terms of the accuracy-complexity trade-off; see supplementary materials S1 Appendix) for fitting the observed responses.”’

typos:

Thank you, we have corrected these typos.

page 7 - should the "T" in T-value and T-test be lower case?

bottom of page 9, line 223, "where chosen" -> "were chosen"

line 357, pg 15: "observed a significant reduced" -> "observed a significant reduction"

We hope these revisions are what you and the editor had in mind.
